# Evaluation of Pharmacy Inquiries in Physician Order Reviews for Medication Safety: A Cross-Sectional Study

**DOI:** 10.3390/medicina58091297

**Published:** 2022-09-16

**Authors:** Jungwon Cho, Koenhee Kim, Young Mi Jeong, Euni Lee

**Affiliations:** 1 Research Institute of Pharmaceutical Sciences & College of Pharmacy, Seoul National University, Seoul 08826, Korea; 2 Department of Pharmacy, Seoul National University Bundang Hospital, Seongnam-si 13620, Korea

**Keywords:** medication use process, medication error, drug-related problems, pharmacy intervention, health information systems, pharmaceutical care

## Abstract

Background and Objectives: Despite the effort to prevent drug-related problems (DRPs) in healthcare settings, prescribing errors are common in the medication use process. In a Korean teaching hospital, pharmacists verify prescription orders during their routine order review process and document the details in a homegrown health information system (HIS). The objectives of this study were to identify the annual trends in pharmacy inquiries and to evaluate the prevalence of the inquiries by drug ingredients, including a description of the “pharmacy inquiry” screen in the HIS. Materials and Methods: A retrospective cross-sectional study was conducted to describe pharmacy inquiries related to preventing potential DRPs during order reviews and to evaluate the associated factors for discontinuation of prescription orders on medication among inquiries using data from January 2008 to December 2021. A descriptive analysis was performed using 128,188 inquiries, documented by 245 pharmacists for 14 years. Results: The frequency of inquiry steadily increased annually. The most frequent cause was “inappropriate dose or regimen” (49.1%) and “piperacillin and beta-lactamase inhibitor” was the most mentioned drug ingredient in the inquiries (3.4%). The overall acceptance rate of the pharmacists’ recommendation was 82.4%, and the cause of the highest acceptance was “inappropriate mix solution” (96.5%). Hospitalization and certain inquiry topics were significantly associated with discontinuation of prescription orders on inquired medications by clinicians. Conclusions: The findings indicate that pharmacy inquiries with integrated HIS could resolve inaccuracy during physicians’ order reviews and ensure safe patient care. As a tool for preventing prescribing errors, the pharmacy inquiry data can help maximize consistent improvement and optimize the medication use process in healthcare settings.

## 1. Introduction

Drug-related problems (DRPs) are the main cause of interference with desired health outcomes during the medication use process in healthcare settings globally [1,2]. “Medication errors,” a major cause of DRPs, can be commonly found in all stages of the medication use process comprising: (1) prescribing; (2) transcribing and documenting; (3) dispensing; (4) administering; and (5) monitoring [3,4]. As medication errors could lead to serious harm, increased length of hospitalization, and even death [5,6], pharmacists have made efforts to enhance treatment effectiveness and safety by providing medication information and clarifying prescriptions during order reviews.

Among the medication errors, prescribing errors can occur during the process of selecting a drug, dose, dosage form, and treatment duration [7]. Instead of handwriting prescriptions, healthcare professionals employed computerized prescriber order entry (CPOE) [8] and established a clinical decision support (CDS) system in a health information system (HIS) to prevent errors by providing drug information and eventually guiding them to the right medication [9]. As these systems cannot eliminate prescribing errors in healthcare settings, pharmacists conduct comprehensive order reviews and consult clinicians for clarifications [10]. Pharmacists not only recommend changes in the orders which concern DRPs but also discuss the patients’ medication therapy plan or feedback information obtained from the patients [11,12].

Ward physicians in teaching hospitals are physicians-in-training who graduated from medical school in the last few years. In such an environment, similar prescribing errors tend to be repeated by new ward physicians during periodic changes. Thus, the role of pharmacy inquiries in prescribing errors is essential to prevent potential DRPs from interfering with the treatment effectiveness or safety. Then, the pharmacists document details of the inquired orders, including their causes, recommendations, and results. In documenting pharmacy inquiries, HIS could be utilized to support pharmacists’ work performance and enhance communication among healthcare professionals. Moreover, detailed HIS documentation of pharmacy inquiries is a provision of a written record and subsequent retrieval for further training within the pharmacy department [13]. Although better understanding and utilization of this database is linked to improving clinical services in healthcare settings, related research is limited.

Therefore, this study aimed to analyze pharmacy inquiries in HIS to prevent repetitive DRPs and explore quality improvement items for HIS enhancement. This study will describe the data of pharmacy inquiries during order reviews over a period of 14 years, which were accumulated in a teaching hospital. The objectives included identifying the trends and prevalence by drug ingredients in pharmacy inquiries and to evaluate the associate factors for actual discontinuation of inquired medications by clinicians.

## 2. Materials and Methods

### 2.1. Study Site

The Seoul National University Bundang Hospital (SNUBH) is a 1335-bed academic teaching hospital that first opened as a fully digitized hospital using a homegrown HIS known as BESTCare^®^ in 2003. The Healthcare Information and Management Systems Society in the United States certified SNUBH as Stage 7, the highest stage of certification offered by them. The hospital pharmacists at SNUBH monitored patients’ treatment courses through HIS record and periodic visits of patients at different wards, e.g., intensive care units, multidisciplinary nutrition teams, and specific clinical general wards. The dedicated pharmacists performed physician order reviews as a routine clinical practice. During the review process, pharmacy inquiries were generated. 

### 2.2. Study Design and Pharmacy Inquiries as a Data Source

This study was a retrospective cross-sectional study using pharmacy inquiries during the 14-year period from 1 January 2008 to 31 December 2021. 

**Figure 1:**
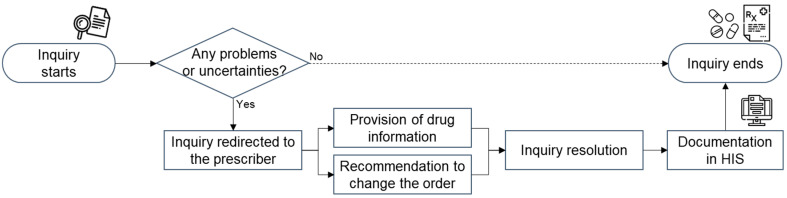
The inquiry process during order review in the pharmacy department (icons made by Freepik [https://www.freepik.com, accessed on 15 July 2022] from www.flaticon.com, accessed on 15 July 2022). HIS: health information system.

**Figure 2:**
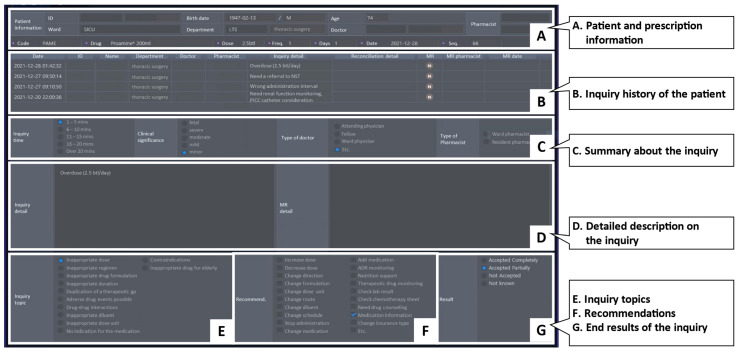
A captured screen of “pharmacy inquiry” in the homegrown health information system, BESTCare^®^, was displayed and all contents in the screen have been translated into English for publication. The screen enables the pharmacists to document detailed inquiries, such as patient and prescription information, inquiry history of the patient, summary of the inquiry including time, type of clinician, detailed description on the inquiry, inquiry topics, recommendations, and end results of the inquiry. The pharmacy inquiry is stored in the electronic database and is shared by all pharmacists for further patient-centered care.

In this study, the term “pharmacy inquiry” was defined that pharmacists identify potential DRPs or provide medication information to the prescribing physicians during order reviews. The inquiry process (Figure 1) begins with the pharmacist reviewing the orders by checking whether the right medication was selected and correctly prescribed in terms of the dose, frequency, or treatment duration regarding a patient’s status, such as present illness or renal function. If prescription errors or uncertainties are detected during this step (e.g., dose adjustment for decreased renal clearance in older people), pharmacists consult clinicians about the prescription and communicate with physicians on the phone, via electronic messenger, or face-to-face meetings. The inquiry topics are classified into two types: (1) provision of drug information and (2) recommendation for a change in the patient’s medication therapy. Until the purpose of the inquiry is fulfilled, the pharmacist communicates with the clinician. Finally, the pharmacist documents the inquiry details on a HIS screen. All pharmacists in SNUBH can access this screen for further order reviews as the inquiry records are stored for each specific patient. The screen layout of the pharmacy inquiry is described in Figure 2. The screen aimed to focus on patient-centered medication profiles by documenting and sharing previous inquiries, such as the inquiry history of the patient, inquiry summary and detailed description, topics, causes or recommendations, and the end results of the inquiry.

The data included pharmacy inquiries about patients who were hospitalized or admitted to SNUBH and were collected from SNUBH BESTCare^®^.

### 2.3. Outcomes

The primary outcomes were to identify annual trends in the pharmacy inquiries by inquiry type and to evaluate the prevalence of the inquiries by drug ingredients. A total of 22 inquiry topics were classified into two types: (1) provision of drug information and (2) recommendation for a change in the patient’s medication therapy. We focused on the second type, which comprised 12 subcategories as described in Table 1. Specifically, the 12 inquiry topics were inappropriate orders in dose or regimen, dose unit, drug form/formulation, duration of treatment, administration route, diluent; inappropriate medications for the elderly; duplication of a therapeutic group/ingredient; possible adverse drug events; no indication for the medication; drug–drug interactions; contraindications. The data on the most frequently mentioned drug ingredients were also estimated for every subcategory of causes. Drug ingredients were collected using the 5th level of the Anatomical Therapeutic Chemical (ATC) classification system by the World Health Organization Collaborating Center. 

In addition to the trends and prevalence mentioned above, we analyzed the end result of acceptance for pharmacists’ recommendations. At the last step of inquiries, the pharmacist recommends more appropriate medication to the clinicians than their current prescription, e.g., change of dose, frequency, route, or duration. If the clinician accepts the recommendation completely or partially, that inquiry is recorded as “accepted completely” or “accepted partially” by the pharmacist on the HIS screen. In case of clinician elected not to accept the recommendation, pharmacist made communication with the clinician, mostly via telephone or electronic messenger, the inquiry was recorded as “not accepted.” Although our current information system does not have menu to select the reason for “non accepted,” most of the clinicians conveyed that “therapeutic benefits outweigh the risks due to the medications” as their reasons for non-acceptance.

Lastly, we evaluated the associate factors for discontinuation of prescription orders on medication among inquiries by clinicians. The information indicating a discontinuation of the prescription order was electronically collected from HIS as the proxy indicator to change or stop prescription orders after inquiries were made.

### 2.4. Statistical Analysis

The study analysis focused on describing the prevalence of the inquiry type and drug ingredients in the pharmacy inquiry data. Descriptive statistics were performed to summarize the number of the inquired prescription orders, list frequently mentioned medications, and report acceptance rates of the inquiries by inquiry topics. Multivariable logistic regression analysis was conducted to assess factors for discontinuation of prescription orders on inquired medication (yes/no), set as the dependent variable. The adjusted odds ratios (ORs) with 95% confidence intervals were determined while adjusting confounders such as visit type, department, and inquiry topics. All statistical analyses were performed using R version 4.0.2 2020 (The R Foundation for Statistical Computing, Vienna, Austria).

## 3. Results

### 3.1. Trends in Pharmacy Inquiry

**Figure 3:**
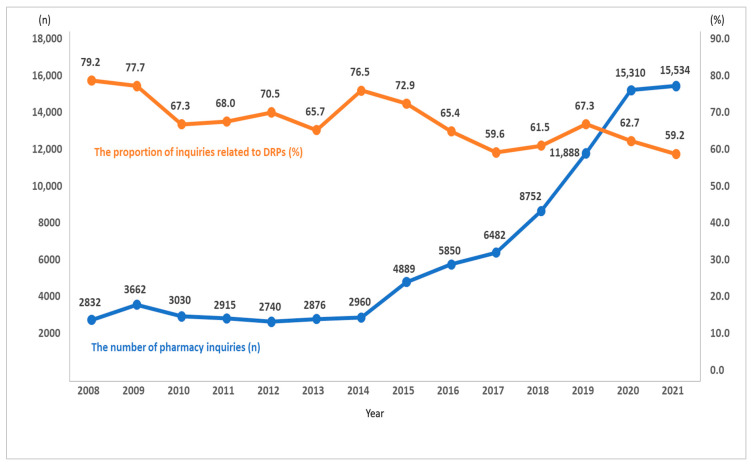
The number of pharmacy inquiries (blue line, *n*) and the proportion of inquiries related to DRPs (orange line, %). DRPs: drug-related problems.

During the 14-year study period, 245 pharmacists documented 128,188 inquiries. Among them, 12 subcategories of inquiry topics (inquiries related to preventing potential DRPs) amounted to 58,630 (45.7%). Figure 3 shows the annual number of pharmacy inquiries and the proportion related to preventing potential DRPs. Although the number of pharmacy inquiries has steadily increased, the proportion of inquiries related to DRPs has decreased (Figure 3).

### 3.2. Overall Characteristics of Pharmacy Inquiries

Overall characteristics of pharmacy inquiry data are presented in Table 1. The most frequently inquired topics were “inappropriate dose or regimen” (28,782, 49.1%) and “inappropriate drug formulation” (8508, 14.5%). The former comprised drug doses too low or high and regimens too frequent or not frequent enough. The latter included topics for formulation choices in case specific drug ingredients were produced with several formulations, for instance, capsule, tablet, or injection. The inquiry topic of “adverse drug events possible” was related to preventing adverse drug events based on the approved drug labels by the Ministry of Food and Drug Safety in Korea, e.g., aspirin plain tablet instead of enteric capsule to the patient with peptic ulcer, or standard dose to the patients on hemodialysis. All 12 subcategories of topics are shown in Table 1.

### 3.3. Frequently Mentioned Medications

The frequently mentioned medications in pharmacy inquiries are presented in Table 2. The most frequently mentioned drug ingredients related to preventing DRPs were antibacterials for systemic use, such as “piperacillin and beta-lactamase inhibitors” (1997, 3.4%), “levofloxacin” (1381, 2.4%), “ciprofloxacin” (1251, 2.1%), or “vancomycin” (873, 1.5%). Since those antibiotics were used in various formulations and potencies in the hospital, 14 antibiotic ingredients were frequently inquired about. The pharmacy inquiries for gastrointestinal medications such as “famotidine” and “pantoprazole” were highly ranked, with 1482 (2.5%) and 914 (1.6%) pharmacy inquiries, respectively. The rank of drug ingredients has been changed during the 14-year study period. For instance, the ingredients of “blood substitutes and perfusion solutions” were highly ranked for initial period, however, decreased after 2012, and the “antibacterials for systemic use” were increased steeply after 2014.

### 3.4. Result of Acceptance for Pharmacists’ Recommendations

Table 3 presents the result of recommendations for each of the 12 subcategories of inquiry topics. The mean acceptance rate of total inquiries was 82.4% (48,295 out of 58,630 inquiries). Regarding the causes, “inappropriate mix solution” (96.5%, e.g., the maximum concentration of rituximab, incompatible amphotericin B with normal saline), “inappropriate dose unit” (91.1%, e.g., piperacillin/tazobactam 4.45 vial instead of 4.45 g [1 vial contains piperacillin 4 g and tazobactam 0.45 g]), and “adverse drug events possible” (90.7%) had the highest acceptance rate. Meanwhile, “inappropriate administration route” (63.9%, e.g., proposal intravenous to oral levofloxacin), “contraindications” (76.8%, e.g., antitussive syrup to infants), and “no indication for the medication” (77.4%, e.g., preventive use of broad-spectrum without blood culture) had the lowest acceptance rate. 

### 3.5. Associated Factors for Discontinuation of Prescription Orders on Medication among Inquiries

Several characteristics were found to be associated with discontinuation of prescription orders on medication among inquiries by clinicians (Table 4). With respect to visit type, inquiries about patients who were hospitalized to general wards (aOR = 1.060, 95% CI 0.994–1.131) and intensive care units (aOR = 1.227, 95% CI 1.135–1.327) had increased probabilities of discontinuation compared to the patients who were admitted to ambulatory clinics. Regarding inquiry topics, inquiries of “inappropriate dose unit” (aOR = 4.353, 95% CI 3.683–5.164), “inappropriate diluent“(aOR = 2.248, 95% CI 1.986–2.547), and “inappropriate dose or regimen“ (aOR = 1.823, 95% CI 1.690–1.967) were more likely to discontinue prescription orders compared to inquiries of “no indication for the medication”.

## 4. Discussion

This study showed the trends of pharmacy inquiries, an evaluation of their prevalence by drug ingredients, and associated factors for discontinuation of prescription orders on medication among inquiries. The significance of our findings is three-fold: First, a meaningful clinical contribution was made by accumulating real-world data from pharmacy practice over 14 years. The inquiry data formed a valuable database that could serve clinical practice in a healthcare institution since pharmacy inquiries in homegrown HIS have been continuously well managed by the pharmacists. Furthermore, the pharmacists at SNUBH reviewed physicians’ orders and documented their inquiries in a precise and detailed manner to prevent potential DRPs. The construction and utilization of the inquiry data were feasible through the leadership of the pharmacy department and the collaborative medical informatics team in SNUBH. Second, it was found that the clinicians accepted the pharmacists’ recommendations at a high average rate. Although the total pharmacy inquiries continuously increased, the acceptance rate was sustained by over 80%. This rate indicates that the role of pharmacists was important for preventing potential DRPs in the medication use process. Thirdly, multivariable logistic regression analysis showed that hospitalization at intensive care units compared to ambulatory clinics, certain departments, and inquiry topics were significantly associated with discontinuation of prescription orders. Among 12 inquiry topics, topics related to safety issues such as dose or regimen, drug form/formulation, and dose unit were highly associated with discontinuation of prescription orders. 

During the 14-year period, the pattern of frequently inquired medication types were slightly changed related to new clinical settings or HIS modifications at the SNUBH. We believe that the inquiries about the “blood substitutes and perfusion solutions” were decreased when the new CPOE system for total nutrition admixture was developed for ward physicians who prescribed in a neonatal intensive care unit in 2012. Since the new system guided appropriate dose/regimen for neonatal patients, the rate of prescribing errors was decreased. The increase in the inquiries about the “antibacterials for systemic use” was appeared to be consistent with our SNUBH system changes made in 2014 by allocating a designated pharmacist for safeguarding the optimal antibiotics use. The antibiotics pharmacist has been conducting intensive monitoring of empiric antibiotics use, preventing antibiotics abuse, and proposing intravenous antibiotics to oral formulations, if possible. Thus, the total inquiries by antibiotic pharmacists were highly frequent. The clinical role of pharmacists has evolved due to the increase in the number of prescriptions annually and the multidisciplinary patient care, e.g., comprehensive geriatric care team, antimicrobial stewardship program, and nutrition support team. Therefore, pharmacists’ order reviews related to preventing potential DRPs or providing medication information have increased [6]. As the previous study suggests the value of engaging pharmacists in checking medication orders [14], the demand for convenient health information technology (HIT) has increased to relieve the burden of pharmacy manpower. A step forward can be taken by strategically improving HIT by utilizing screens with integrated information to improve the provision of education or clinical practice. 

The results showed that “inappropriate dose or regimen” comprised approximately 50% (28,782) of the 58,630 inquiries. As this study derived the frequency, acceptance rate, and drug ingredients of pharmacy inquiries comprehensively in the real-world practice setting, frequently inquired causes were clinically meaningful. The use of HIT led to benefits in medication orders. However, certain types of medication errors could occur due to the improper use of HIT [15]. Despite the implementation of HIT to enhance patient safety, specifically, the CDS system in BESTCare^®^, the selection of drugs with inappropriate dosages or regimens was predominantly high. This indicates the possibility of an error that the CDS system cannot overcome; therefore, pharmacists continue to play an important role in the process of medication use. For example, the gastrointestinal medications such as H_2_-receptor antagonists are one of the frequently prescribed medications requiring adjustments on their doses or regimens based on patient’s kidney function. Second, as the cause of “drug form/formulation” was the highest proportion of the inquiries (8508 out of 58,630 inquiries), the expertise of pharmacists was critical to help clinicians choose the most appropriate formulation in the SNUBH formulary system considering patients’ profiles, such as the route of administration. Selection of the most proper formulation was essential to achieve optimal drug efficacy and minimize adverse drug events. We believe that our findings from the medication inquiries could help resolve this inaccurate dose/regimens/formulation. Consequently, pharmacy inquiries with integrated HIT could prevent potentially harmful DRPs and improve patient safety. Furthermore, the pharmacy inquiry data can be considered as a tool for subsequent retrieval for training within the pharmacy department. The SNUBH currently fosters in-hospital education program to prevent medication errors using pharmacy inquiries at semi-annual workshops for clinicians, monthly seminars for all healthcare professionals. The pharmacists also plan education programs to improve CPOE system with an ultimate goal of reducing medication errors.

This study has some limitations. First, the pharmacists’ subjectivity might influence the process of classifying the inquiry topics because the career or training backgrounds of the pharmacists were quite diverse. However, as the senior pharmacists have established internal manuals and periodic seminars for the pharmacists to follow a standardized inquiry process, we believed the potential influence of subjectivity in the classification of the inquiry topics would be minimal. Second, we only analyzed the total number of inquired medications by ingredients. We could not collect the proportion of prescriptions that contains inquired medications. Therefore, further study may be needed to address the detailed proportion of inquired medications over all prescription orders. Thirdly, although this study reported the result of acceptance for pharmacists’ recommendation for inquiry topics, clinical outcomes for inquiries such as length of hospital stay, mortality, and occurrence of adverse drug events could not be evaluated. However, we believe that the acceptance of the recommendation could serve as a proxy indicator for preventing potential DRPs. As a well-structured HIT plan for advanced pharmaceutical care, we have planned the development of machine learning algorithms for detecting abnormal or inappropriate medication prescriptions based on previous inquired medications by the pharmacists. 

## 5. Conclusions

Our findings highlight the description of the pharmacy inquiries as a part of pharmacy intervention during order reviews and evaluation of the prevalence of inquiries by drug ingredients in the long-term period. The highest drug ingredients in pharmacy inquiries were antibacterials for systemic use. The highly accepted inquiry topics were “inappropriate mix solution” and “inappropriate dose unit”. Pharmacy inquiries steadily increased and the overall acceptance rate for pharmacists’ recommendations was sustained. Hospitalization at intensive care units, certain departments, and inquiry topics were significant factors associated with discontinuation of prescription orders on medication among inquiries. This study provides a well-structured HIS screen with pharmacists’ practice during the process of physician order entry including components of the inquiry and documentation panes for the clinicians, which could be adaptable to other hospital settings.

## Figures and Tables

**Table 1:** Overall characteristics.

Characteristics	Categories	*n* (%)
Visit type	Hospitalization—general wards Hospitalization—intensive care units Ambulatory clinics Emergency department	41,855 (71.4) 8356 (14.3) 4869 (8.3) 3550 (6.1)
Department	Internal medicine Surgery Emergency Orthopedics Pediatrics Neurology Obstetrics and gynecology Urology Neuropsychiatry Others ^1^	27,918 (47.6) 9270 (15.8) 4481 (7.6) 4112 (7.0) 3695 (6.3) 3680 (6.3) 1287 (2.2) 1013 (1.7) 627 (1.1) 2547 (4.3)
Inquiry topics (causes to change the order)	Inappropriate dose or regimen Inappropriate drug form/formulation Inappropriate duration of treatment Duplication of a therapeutic group/ingredient Adverse drug events possible No indication for the medication Drug-drug interactions Inappropriate diluent Inappropriate administration route Inappropriate dose unit Contraindications Inappropriate medications for elderly	28,782 (49.1) 8508 (14.5) 3807 (6.5) 3335 (5.7) 3051 (5.2) 3019 (5.1) 2112 (3.6) 1711 (2.9) 1489 (2.5) 977 (1.7) 932 (1.6) 907 (1.5)

^1^ Others included ophthalmology, dermatology, anesthesiology, and radiation oncology.

**Table 2:** Top 30 frequent drug ingredients of pharmacy inquiries.

Drug Ingredients		Pharmacy Inquiries		
3rd Level of the ATC * Codes	5th Level of the ATC * Codes	Rank	*n*	%
Antibacterials for systemic use	Piperacillin and beta-lactamase inhibitor Levofloxacin Ciprofloxacin Vancomycin Cefadroxil Sulfamethoxazole and trimethoprim Metronidazole Amoxicillin and beta-lactamase inhibitor Cefazolin Ceftriaxone Colistin Meropenem Ertapenem Ampicillin and beta-lactamase inhibitor	1 3 4 7 8 9 12 13 16 19 20 22 23 30	1997 1381 1251 873 813 789 737 736 655 569 553 490 481 443	3.4 2.4 2.1 1.5 1.4 1.3 1.3 1.3 1.1 1.0 0.9 0.8 0.8 0.8
Drugs for acid-related disorders	Famotidine Pantoprazole Esomeprazole Lansoprazole	2 6 25 28	1482 914 455 448	2.5 1.6 0.8 0.8
Antithrombotic agents	Acetylsalicylic acid	5	1126	1.9
Blood substitutes and perfusion solutions	Intravenous solutions Sodium chloride	10 18	775 631	1.3 1.1
Analgesics	Paracetamol (acetaminophen) Tramadol and paracetamol	11 17	746 635	1.3 1.1
Drugs used in diabetes	Metformin Sitagliptin	14 27	684 449	1.2 0.8
General nutrients	Carbohydrates	15	666	1.1
Cough and cold preparations	Hederae helicis folium Ambroxol	21 26	491 453	0.8 0.8
Vitamins	Multivitamins, plain	24	469	0.8
Calcium channel blockers	Amlodipine	28	448	0.8

* ATC: Anatomical therapeutic chemical.

**Table 3:** Acceptance rates of pharmacists’ recommendations by inquiry topics.

Inquiry Topics	Total Inquiries (*n*)	Accepted Inquiries (*n*)	Acceptance Rate (%)
Inappropriate diluent Inappropriate dose unit Adverse drug events possible Drug-drug interactions Inappropriate medications for elderly Inappropriate drug form/formulation Duplication of a therapeutic group/ingredient Inappropriate duration of treatment Inappropriate dose or regimen No indication for the medication Contraindications Inappropriate administration route	1711 977 3051 2112 907 8508 3335 3807 28,782 3019 932 1489	1651 890 2767 1906 783 7191 2790 3141 23,171 2338 716 951	96.5 91.1 90.7 90.2 86.3 84.5 83.7 82.5 80.5 77.4 76.8 63.9

**Table 4:** Associated factors for discontinuation of prescription orders on medication.

Characteristics		Adjusted OR	95% CI
Visit types	Ambulatory clinics Hospitalization—general wards Hospitalization—intensive care units Emergency department	1.000 (reference) 1.060 1.227 * 0.647 *	0.994–1.131 1.135–1.327 0.546–0.766
Departments	Internal medicine Surgery Emergency Orthopedics Pediatrics Neurology Neuropsychiatry Obstetrics and gynecology Urology Others ^1^	1.000 (reference) 0.932 * 1.633 * 0.801 * 1.049 0.698 * 0.630 * 1.006 0.795 * 1.443 *	0.887–0.979 1.416–1.888 0.749–0.857 0.976–1.127 0.650–0.750 0.535–0.741 0.897–1.129 0.700–0.903 1.320–1.579
Inquiry topics	No indication for the medication Inappropriate dose or regimen Inappropriate drug form/formulation Inappropriate duration of treatment Duplication of a therapeutic group/ingredient Adverse drug events possible Drug-drug interactions Inappropriate diluent Inappropriate administration route Inappropriate dose unit Contraindications Inappropriate medications for elderly	1.000 (reference) 1.823 * 1.642 * 1.260 * 1.152 * 0.718 * 0.519 * 2.248 * 0.671 * 4.353 * 1.361 * 0.796 *	1.690–1.967 1.510–1.787 1.145–1.388 1.042–1.272 0.647–0.796 0.461–0.584 1.986–2.547 0.590–0.763 3.683–5.164 1.174–1.578 0.684–0.927

* *p* < 0.05; OR: odds ratio; CI: confidence interval. ^1^ Others included ophthalmology, dermatology, anesthesiology, and radiation oncology.

## Data Availability

Data sharing is not applicable to this article.
